# Self‐gated, dynamic contrast‐enhanced magnetic resonance imaging with compressed‐sensing reconstruction for evaluating endothelial permeability in the aortic root of atherosclerotic mice

**DOI:** 10.1002/nbm.4823

**Published:** 2022-09-23

**Authors:** Claudia Calcagno, John A. David, Abdallah G. Motaal, Bram F. Coolen, Thijs Beldman, Alexandra Corbin, Arnav Kak, Sarayu Ramachandran, Alison Pruzan, Arthi Sridhar, Raphael Soler, Christopher M. Faries, Zahi A. Fayad, Willem J. M. Mulder, Gustav J. Strijkers

**Affiliations:** ^1^ Biomedical Engineering and Imaging Institute Icahn School of Medicine at Mount Sinai New York USA; ^2^ Department of Diagnostic, Molecular and Interventional Radiology Icahn School of Medicine at Mount Sinai New York USA; ^3^ Amsterdam University Medical Centers, Department of Medical Biochemistry, Amsterdam Cardiovascular Sciences University of Amsterdam Amsterdam The Netherlands; ^4^ Siemens Healthineers, Cardiovascular Care Group, Advanced Therapies Business Erlangen Germany; ^5^ Amsterdam University Medical Centers, Department of Biomedical Engineering and Physics, Amsterdam Cardiovascular Sciences University of Amsterdam Amsterdam The Netherlands; ^6^ Department of Internal Medicine Radboud University Medical Center Nijmegen The Netherlands; ^7^ Radboud Institute for Molecular Life Sciences Radboud University Medical Center Nijmegen The Netherlands; ^8^ University of Texas Southwestern Medical Center Dallas TX USA; ^9^ Department of Hematology/Oncology UTHealth McGovern Medical School Houston TX USA; ^10^ CNRS, CRMBM Marseille France; ^11^ Department of Vascular and Endovascular Surgery Hôpital Universitaire de la Timone, APHM Marseille France

**Keywords:** atherosclerosis, DCE‐MRI, inflammation, microvascularization, mouse, self‐gated

## Abstract

High‐risk atherosclerotic plaques are characterized by active inflammation and abundant leaky microvessels. We present a self‐gated, dynamic contrast‐enhanced magnetic resonance imaging (DCE‐MRI) acquisition with compressed sensing reconstruction and apply it to assess longitudinal changes in endothelial permeability in the aortic root of Apoe^−/−^ atherosclerotic mice during natural disease progression. Twenty‐four, 8‐week‐old, female Apoe^−/−^ mice were divided into four groups (n = 6 each) and imaged with self‐gated DCE‐MRI at 4, 8, 12, and 16 weeks after high‐fat diet initiation, and then euthanized for CD68 immunohistochemistry for macrophages. Eight additional mice were kept on a high‐fat diet and imaged longitudinally at the same time points. Aortic‐root pseudo‐concentration curves were analyzed using a validated piecewise linear model. Contrast agent wash‐in and washout slopes (*b*_*1*_ and *b*_*2*_) were measured as surrogates of aortic root endothelial permeability and compared with macrophage density by immunohistochemistry. *b*_*2*_, indicating contrast agent washout, was significantly higher in mice kept on an high‐fat diet for longer periods of time (*p* = 0.03). Group comparison revealed significant differences between mice on a high‐fat diet for 4 versus 16 weeks (*p* = 0.03). Macrophage density also significantly increased with diet duration (*p* = 0.009). Spearman correlation between *b*_*2*_ from DCE‐MRI and macrophage density indicated a weak relationship between the two parameters (r = 0.28, *p* = 0.20). Validated piecewise linear modeling of the DCE‐MRI data showed that the aortic root contrast agent washout rate is significantly different during disease progression. Further development of this technique from a single‐slice to a 3D acquisition may enable better investigation of the relationship between in vivo imaging of endothelial permeability and atherosclerotic plaques' genetic, molecular, and cellular makeup in this important model of disease.

## Graphical abstract

Atherosclerotic plaques at high risk of causing cardiovascular events are characterized by a prominent inflammatory infiltrate accompanied by the presence of an extensive network of leaky microvessels. In this paper, we present a dynamic contrast‐enhanced magnetic resonance imaging (DCE‐MRI) acquisition and compressed sensing reconstruction to aortic root in mouse models of atherosclerosis. In a longitudinal study, we find that the contrast agent washout slope, calculated from this acquisition, is significantly different during the disease natural progression.

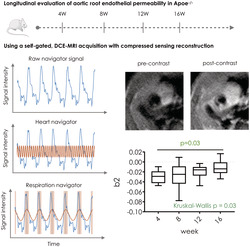

**Abbreviations used**
AIF: arterial input function
AUC: area under the curve
CS: compressed sensing
DCE‐MRI: dynamic contrast‐enhanced magnetic resonance imaging
ECG: electrocardiogram
FLASH: fast low angle shot
GRASP: Golden‐angle RAdial Sparse Parallel
TE: echo time
TR: repetition time

## 1 INTRODUCTION

Cardiovascular disease due to atherosclerosis is the leading cause of morbidity and mortality worldwide.^1^ Dysfunction and increased permeability of the vascular endothelium are crucial for the initiation and progression of atherosclerosis.^2^ In the initial stages of disease, a leaky luminal endothelium allows the translocation and accumulation of circulating low‐density lipoproteins into the artery's tunica intima. This phenomenon triggers a maladaptive immune response that results in the infiltration of inflammatory cells into the arterial wall, plaque formation and, eventually, the possible onset of severe clinical events, such as myocardial infarction and stroke.

Much effort has been devoted to developing and validating in vivo quantitative imaging techniques to measure plaque permeability and microvascularization as markers of atherosclerotic plaque vulnerability, both in humans^3^, ^4^, ^5^ and in large animal models of atherosclerosis,^6^, ^7^ such as rabbits and pigs.

Compared with larger animal models, mice are less expensive and enable well‐powered longitudinal studies to noninvasively investigate changes in plaque permeability during disease progression, or regression after therapeutic intervention. Secondly, in large animals, in vivo imaging findings are often validated using only histology and immunohistochemistry. On the contrary, nowadays, sophisticated genetic, molecular, and immunological assays are also widely available in mice. This may give us the opportunity to investigate the relationship between imaging metrics of plaque vulnerability, and the genetic, molecular, and cellular makeup of vulnerable atherosclerotic plaques at a more in‐depth level.

Here, we present the development of a self‐gated, dynamic contrast‐enhanced (DCE) magnetic resonance imaging (MRI) acquisition with compressed sensing (CS) reconstruction to quantify endothelial permeability in the mouse aortic root in vivo and noninvasively. DCE‐MRI is an imaging technique that allows measuring of the quantitative parameters related to the tissues' permeability and microvascular volume from the serial, rapid acquisition of images before, during, and after contrast agent injection. We apply and validate our self‐gated DCE‐MRI acquisition in a longitudinal study using genetically modified atherosclerotic Apoe^−/−^ mice, to investigate change in aortic root endothelial permeability during natural disease progression, and its correlation with atherosclerotic plaque inflammatory cell content (macrophages) over time. In the future, we foresee that this methodology may be applied in longitudinal mouse atherosclerosis studies to further characterize disease progression and to evaluate the effect of novel or established antiatherosclerotic compounds on plaque permeability in the mouse model.

## 2 MATERIALS AND METHODS

### 2.1 Study design

**FIGURE 1:**
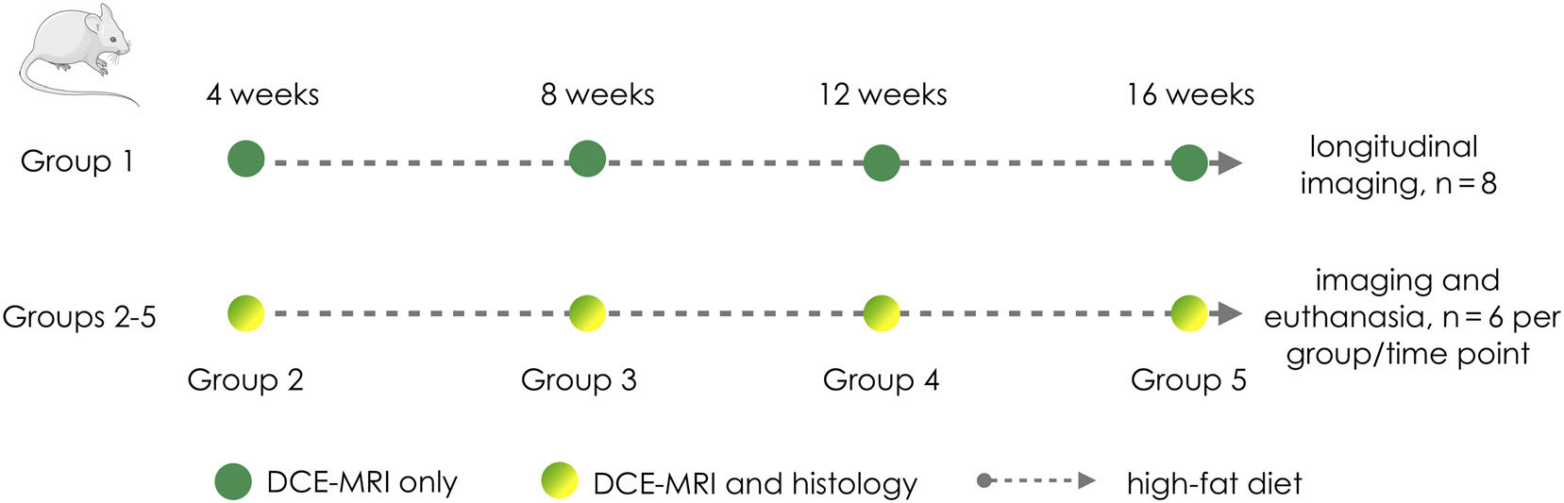
Study design. The study comprised a total of 32 mice, divided into five groups. The first group of eight mice (group 1 at the top), underwent four longitudinal dynamic contrast‐enhanced magnetic resonance imaging (DCE‐MRI) sessions, at 4, 8, 12, and 16 weeks, after initiation of a high‐fat diet. Groups 2–5 were instead used for comparison between DCE‐MRI and histology. Each group consisted of six mice, which underwent imaging and euthanasia respectively at 4, 8, 12, and 16 weeks after high‐fat diet initiation. Green circles indicate DCE‐MRI–only sessions for group 1. Green and yellow circles indicate DCE‐MRI and euthanasia sessions for groups 2–5. The gray dashed arrows indicate maintenance of a high‐fat diet

A total of 32, 8‐week‐old, female Apoe^−/−^ mice, divided into five groups, were used for this study (Figure 1, study design). All mice were fed a high cholesterol diet (TD88137, 42% calories from fat, manufactured by Envigo; http://www.envigo.com). Eight mice in group 1 were used for longitudinal DCE‐MRI sessions at 4, 8, 12, and 16 weeks after high‐fat diet initiation, without final euthanasia. The remaining 24 animals were divided into groups 2–5, each composed of six mice. The mice in groups 2–5 were also imaged, respectively, at 4, 8, 12, and 16 weeks after diet initiation, but, differently than the mice in group 1, were euthanized for ex vivo validation with CD68 immunohistochemistry for macrophages. All animal experiments were performed in accordance with protocols approved by the Institutional Animal Care and Use Committee at the Icahn School of Medicine at Mount Sinai and followed National Institutes of Health guidelines for animal welfare. Data are available from the corresponding author upon reasonable request.

### 2.2 Self‐gated DCE‐MRI: Theory

The mouse aortic root is a vascular territory where vulnerable and permeable atherosclerotic plaques develop consistently and abundantly, which makes it an ideal location for developing in vivo DCE‐MRI. However, there are several challenges to the development of DCE‐MRI in this vascular territory in mice. The aortic root moves constantly because of heart and respiratory motion. Therefore, imaging of this very small structure needs to be synchronized with the cardiac and respiratory cycles. The mouse’s very rapid heart and respiratory rates (typically 500 heartbeats/min and 75 breaths/min during isoflurane anesthesia) preclude the acquisition of an entire high‐resolution and high signal‐to‐noise dynamic image of the root at every single heartbeat. To circumvent these challenges, conventional electrocardiogram (ECG)‐triggered acquisitions usually employ k‐space segmentation, an approach where complete images are reconstructed from the combination of sets of k‐space lines ("segments") acquired over several R‐R intervals, and always in the same phase of the cardiac cycle. This strategy, together with averaging over multiple heartbeats, ensures high signal‐to‐noise acquisitions while minimizing motion artifacts. A drawback of this approach is that prospective gating is rather sensitive to irregular ECG triggers, particularly during the crucial rapid influx of contrast agent when the ECG signal is sometimes temporarily disturbed. An unstable triggering pattern may cause loss of steady‐state signal, and therefore fluctuations in MRI signal weighting, which hampers DCE signal analysis. Additionally, because acquisition timing is dictated by the ECG signals, the time in between subsequent dynamic images may not be constant and may require careful bookkeeping for kinetic data analysis.

**FIGURE 2:**
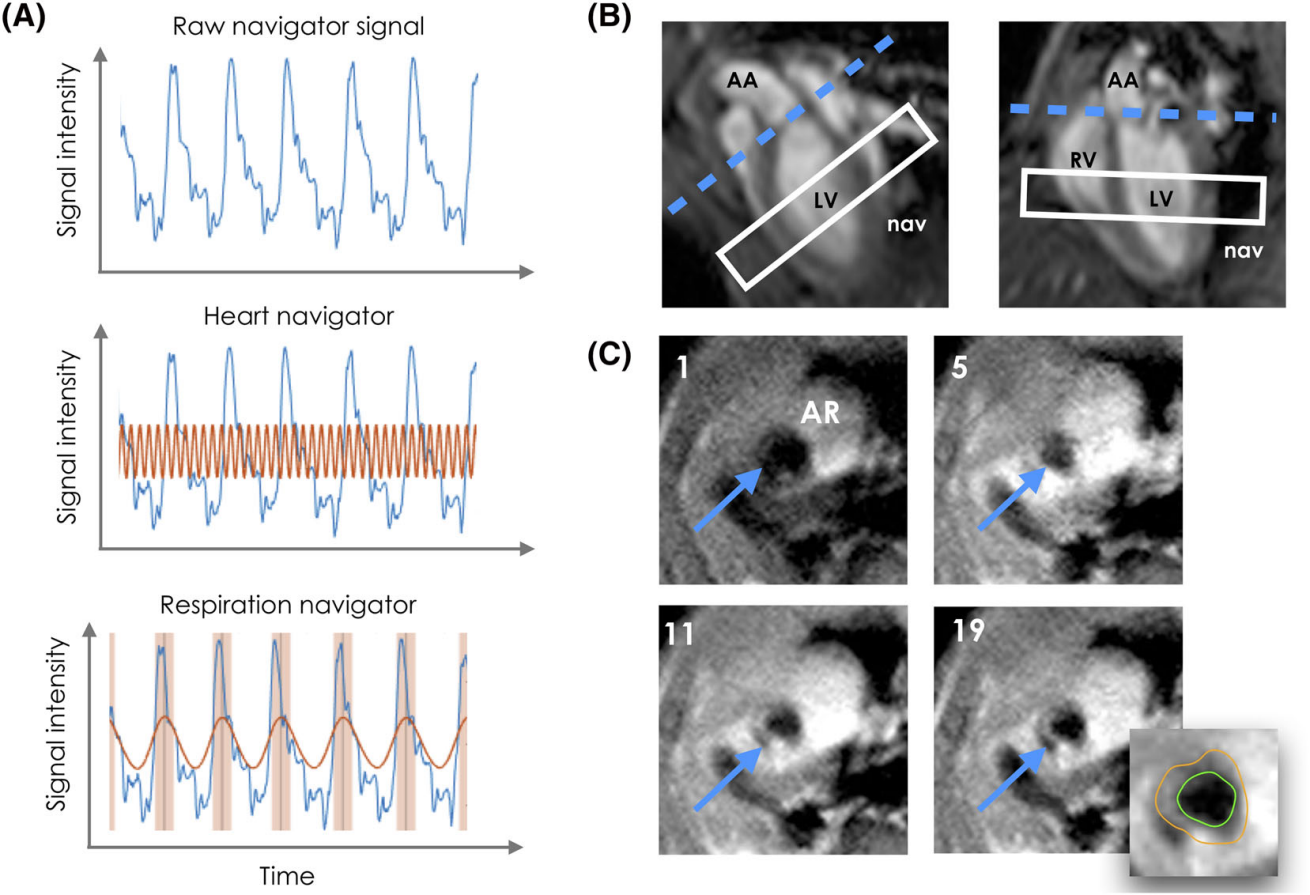
(A) Representative raw, cardiac, and respiratory navigator signal (top, middle, and bottom, respectively). In the middle panel, the red line indicates the cardiac signal extracted from the raw navigator. In the bottom panel, the red line indicates the extracted respiratory signal, while the red shaded areas indicate the detected respiratory motion. (B) Apparent long axis (left) and four‐chamber view (right) used for localizing the aorta root. Blue dashed line, aortic root plane. White rectangle, position of navigator slice. AA, ascending aorta; LV, left ventricle; nav, navigator; RV, right ventricle. (C) Representative images of the aortic root (AR; blue arrow) during the dynamic acquisition, including a representative tracing of the inner (green) and outer (orange) vessel wall counters for frame 19. The number in the top left corner of each panel indicates the corresponding dynamic frame

To overcome these challenges, we propose the novel application of a self‐gated^8^, ^9^, ^10^, ^11^, ^12^, ^13^, ^14^, ^15^, ^16^, ^17^, ^18^ fast low angle shot (FLASH) MRI acquisition (Figure 2) with CS reconstruction for DCE MRI of the aortic root. Self‐gated FLASH acquisitions (either with or without CS reconstructions) are already available on several commercial preclinical MR scanners, where they are commonly referred to as “Intragate” sequences. While Intragate imaging, either with or without CS, is typically used for cine imaging of the moving heart, its application for vascular imaging is novel, and especially for DCE‐MRI.

Unlike ECG‐ and respiratory‐triggered acquisitions, self‐gated FLASH acquisitions rely on a navigator signal for cardiac and respiratory motion compensation. For aortic root imaging, the navigator slice was acquired before each excitation and positioned in the middle of the left ventricle, perpendicular to the cardiac septum. As previously validated for “black blood” cine imaging of the heart,^19^, ^20^ we used a flip angle of 90° to excite the navigator slice. This approach allows saturating the signal from blood flowing out of the left ventricle for better delineation of the aortic root vessel wall.

Because this acquisition is not triggered by external cardiac or respiratory monitors, excitations are applied to the slice or volume of interest continuously (at a time interval defined by the repetition time [TR]), and asynchronously with the cardiac and respiratory cycles, an approach that ensures stable steady‐state signal. In our case, one k‐space line was acquired after each excitation, for a total of 204,800 phase‐encoding steps during a 32 min 25.62 s acquisition (equivalent to 800 repetitions of a matrix consisting of 256 phase‐encoding steps per image).

Because the mouse’s heart rate subtly varies over time and from one cardiac cycle to the next, the same phase‐encoding step (or k‐space line) is almost never measured at exactly the same time in a cardiac cycle. This characteristic feature of the self‐gated sequence ultimately results in the acquisition of a sufficient number of distinct k‐space lines over enough cardiac and respiratory cycles to allow reconstructing images of separate heart phases. In addition, this intrinsically pseudo‐random k‐space filling pattern naturally lends itself to the application of iterative CS algorithms for improved image reconstruction. To fully exploit this feature, our acquisition employed a custom‐designed, weighted k‐space acquisition scheme based on a Gaussian distribution, which heavily oversamples the center of k‐space with respect to the periphery (with center lines constituting 13% of the overall sampling, as opposed to 0.1% for the peripheral lines).

### 2.3 Self‐gated DCE‐MRI: Acquisition

Images were acquired using a Bruker BioSpec 70/30 7‐T preclinical MRI scanner (Bruker, Billerica, MA, USA). After the acquisition of scout images, a single‐slice, self‐gated, cine FLASH acquisition was used to acquire apparent short axis, apparent long axis, and four‐chamber images of the heart (Figure 2B). A single axial slice at the level of the mouse aortic root was planned on the apparent long axis and four chamber images, as shown in Figure 2B (blue dashed line). DCE‐MRI was performed for 32 min using a single‐slice, black blood FLASH acquisition before, during, and after the injection of 0.3 mmol/kg of Gd‐DTPA (Magnevist, Bayer) (Figure 2C). Contrast agent was manually injected via a tail vein catheter 8 min after the start of the acquisition, and was then acquired for an additional 24 min. The imaging parameters were as follows: TR = 9.5 ms; echo time (TE) = 2 ms; flip angle = 10°; pixel size, 100 x 100 μm^2^; slice thickness = 1 mm; field of view, 2.56 x 2.56 cm^2^; matrix size, 256 x 256; number of slices = 1; and number of k‐space trajectory repetitions = 800.

### 2.4 Self‐gated DCE‐MRI: Reconstruction

First navigator signals from the whole acquisition were automatically treated on the scanner for rejection of respiratory events, resulting in approximately 30% of the total k‐space data being eliminated from further processing. Further processing was performed using custom‐made software programmed in Matlab (MathWorks, Natick, MA, USA). Firstly, k‐space and navigator data were separated into 20 temporal frames. A local maximum detection algorithm was then applied to the navigator signal in each temporal frame to identify the start of each cardiac cycle. All detected cardiac cycles were divided into six heart phases. k‐lines acquired within a specific temporal frame were then assigned to these previously determined heart phases depending on their acquisition time within each cardiac cycle. Taking into consideration the approximately 30% of discarded k‐space lines to account for respiratory motion, this resulted in 4.67 averages per cardiac phase in each dynamic frame (average across all lines, although, due to oversampling, the number of averages is respectively higher and lower for central and peripheral lines).

Reconstruction was performed using an iterative CS algorithm to generate high‐quality temporal dynamics of the aortic root without requiring acquisition of the full k‐space matrix.^21^ This approach allowed reconstructing images with combined relatively high spatial (100 x 100 μm^2^) as well as temporal resolution (1 min 36 s), which is necessary to characterize the inflow of contrast agent in the aortic root vessel wall. As mentioned in the previous section, the intrinsic pseudo‐random k‐space filling pattern of self‐gated acquisitions naturally generates incoherent aliasing artifacts, which are necessary to apply CS reconstructions. The requirement for sparsity, which is necessary for CS reconstructions, is also satisfied by the application of a weighted k‐space acquisition matrix. In addition, because only the aortic root and heart are moving, while other tissues and the background are stationary, cine images are sparse in the temporal frequency domain. The aortic root movement during the cardiac cycle is also in itself sparse in the temporal frequency domain, because of the quasi‐periodicity of the heart motion. Last but not least, DCE images are also intrinsically sparse in the temporal dimension, because the same anatomical structures are acquired repeatedly, with only imaging contrast changing over time.

### 2.5 DCE‐MRI analysis

Each DCE‐MRI acquisition was examined by an experienced observer (C.C., 10 years of experience in analyzing vascular images). The cardiac cycle phase was chosen as one where the inner and outer wall were clearly visible in all dynamic frames, with none, or negligible flow artifacts due to poor blood suppression. For this cardiac phase all temporal dynamic frames were traced using Osirix (https://www.osirix-viewer.com). Region‐of‐interest coordinates from tracings performed in Osirix were processed using in‐house Matlab software to extract dynamic curves. Signal intensity was converted to pseudo‐concentration values using the validated linear relationship $C\left(t\right)\propto \frac{\mathrm{SI}\left(t\right)-\mathrm{SI}\left(0\right)}{\mathrm{SI}\left(0\right)}$, where *C(t)* is the contrast agent concentration at time *t*, *SI(t)* is the signal intensity at time *t*, and *SI(0)* is the baseline signal before contrast agent injection. *SI(0)* was calculated as the average signal of frames 2–4 after the start of the acquisition. Pseudo‐concentration curves were analyzed using a previously validated piecewise curve model described by the following set of equations^22^

$$C\left(t\right)=\left\{\begin{array}{c} c\;\forall \;t<\alpha \\ c+b_{1}\left(t-\alpha \right)\;\forall \;\alpha \leq t\leq \beta \\ c+b_{1}\left(\beta -\alpha \right)+b_{2}\left(t-\beta \right)\;\forall \;t>\beta \end{array}\right.,$$

where $C\left(t\right)$ is the contrast agent concentration at time *t*, *c* is the baseline concentration before contrast agent injection, *b*_*1*_ and *b*_*2*_ are respectively the contrast agent washin and washout slopes, while *α* and *β* represent the intersection between the line segments. This approach was used because the arterial input function (AIF), whose knowledge is necessary to apply kinetic modeling, cannot be reliably sampled when using black blood imaging.

### 2.6 Histology

Following imaging, mice were euthanized using sodium pentobarbital and thoroughly perfused. The whole heart and aorta were dissected. The aortic root and arch were embedded in optimal cutting temperature compound and preserved at −80°C. Next, serial 6‐μm thick cross‐sections of the aortic sinus were made using a cryotome (Reichert HistoStat, Cryostat Microtome). From the first cross‐section in which the leaflets of the aortic valves appeared upward, 2–3 serial cross‐sections were obtained, covering the entire aortic sinus area. Sections were stained using CD68 staining to quantify plaque macrophages, using a previously validated protocol.^12^ Aortic sections were digitized using a Panoramic 250 FLASH II digital scanner (3DHISTECH; https://www.3dhistech.com/), at 20 x magnification. After selection of the inner and outer contours of the aortic root using ImageJ (https://imagej.nih.gov/ij/), macrophage‐rich areas were identified in digitized slides using a semiautomated thresholding procedure developed with in‐house Matlab software.^23^ Macrophage density was calculated as the ratio between macrophage‐rich areas and vessel wall area and was expressed as a percentage.

### 2.7 Statistical analysis

Statistical analyses were performed using Prism 7.0a (GraphPad Software, La Jolla, CA, USA; https://www.graphpad.com/). After appropriate tests for normality, changes in DCE‐MRI parameters and macrophages density over time were evaluated using nonparametric Kruskal‐Wallis tests and Spearman correlations. Three datapoints could not be analyzed for DCE‐MRI, while one datapoint could not be analyzed for histology.

## 3 RESULTS

### 3.1 DCE‐MRI

**FIGURE 3:**
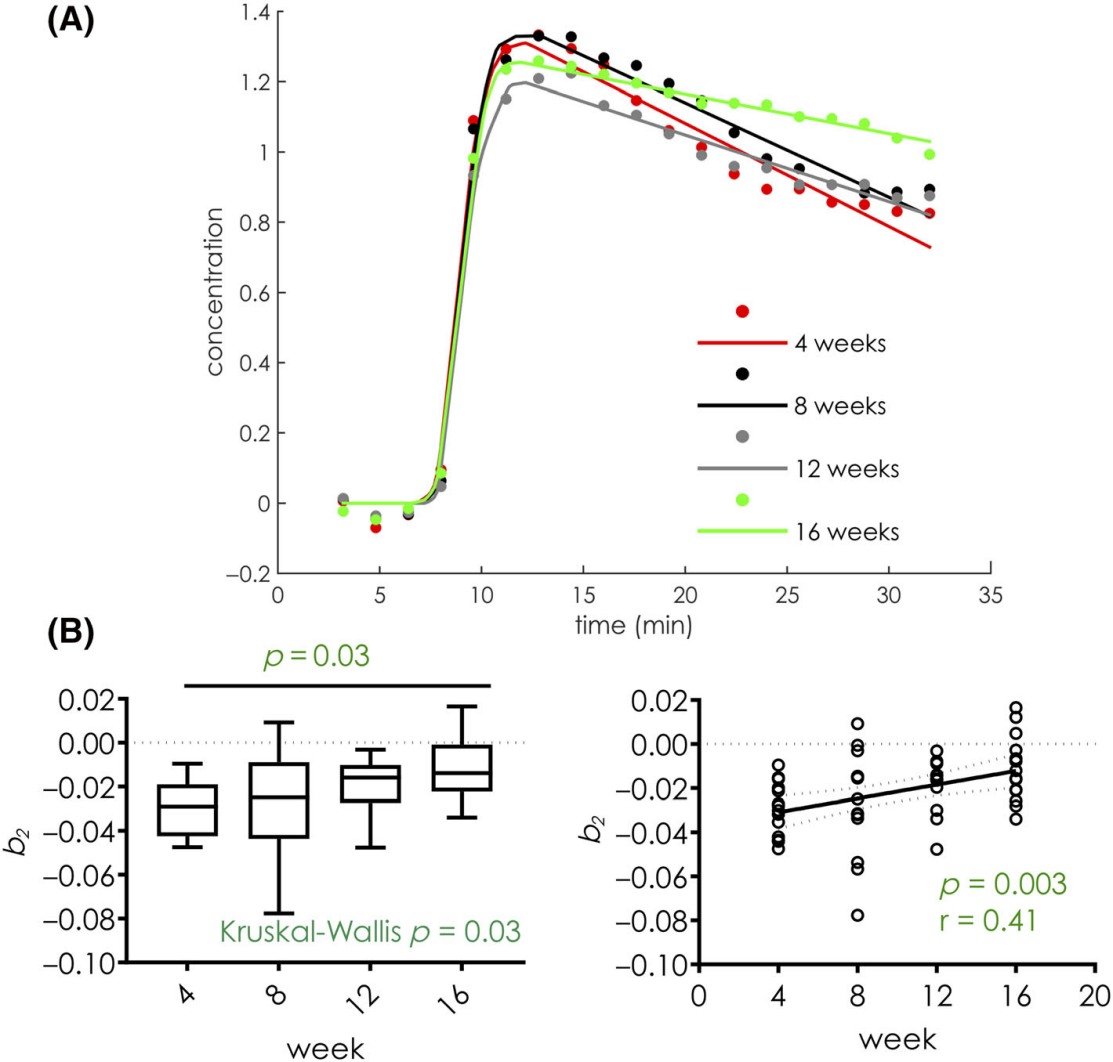
(A) Average curves and fitted curves of mice on 4 (red), 8 (black), 12 (gray), and 16 (green) weeks of a high‐fat diet; from the graph it can be noted that the washout slope *b*_*2*_ becomes less negative as the mice are kept on a high‐fat diet for longer periods of time. (B) Boxplot (left) and linear regression plot (right) of macrophage density in mice 4, 8, 12, and 16 weeks on a high‐fat diet. Midlines in boxplots represent the median, while whiskers represent the minimum and maximum datapoints

No changes in the variables *c* (concentration before contrast agent injection), *b*_*1*_ (contrast agent was in slope), *α* and *β* (intersection between line segments of the piecewise curve) were observed across mice kept on a high‐fat diet for different lengths of time. On the contrary, the variable *b*_*2*_, a measure of the contrast agent washout from the aortic root, was found to be significantly different among different cohorts of mice (Table 1). Figure 3A shows average concentration curves for each group of mice, and the corresponding piecewise curve fit. From the graph it can be observed that the contrast agent washout slope is most negative in the mice kept for 4 weeks on a high‐fat diet, while it progressively approaches zero as the amount of time on the high‐fat diet increases. Comparison of the variable *b*_*2*_ across groups revealed overall significant differences (Kruskal‐Wallis, *p* = 0.03). Side‐by‐side comparison of groups in pairs revealed significant differences between mice on a high‐fat diet for 4 versus 16 weeks (*p* = 0.03) (Figure 3B). Spearman correlation also revealed a significant association between the number of weeks spent on a high‐fat diet and the variable *b*_*2*_ (*p* = 0.003, r = 0.41) (Figure 3B).

**TABLE 1:** Median and 95% confidence intervals (in parentheses) of parameters extracted from piecewise curve modeling at each imaging time point, across mice. The last column represents Kruskal‐Wallis *p* values (significant values are coded in red)

Table 1	4 weeks	8 weeks	12 weeks	16 weeks	*p* value
***b***_***1***_	0.45 (0.27; 0.74)	0.41 (0.36; 0.60)	0.37 (0.30; 0.73)	0.44 (0.22; 0.64)	0.97
***b***_***2***_	−0.03 (−0.04; −0.02)	−0.02 (−0.04; −0.01)	−0.02 (−0.03; −0.00)	−0.01 (−0.02; −0.00)	0.03
***α***	7.69 (7.44; 7.92)	7.77 (7.74; 7.90)	7.80 (7.78; 7.82)	7.76 (7.49; 7.82)	0.34
***β***	10.49 (9.68; 11.40)	10.54 (9.97; 10.85)	10.41 (9.75; 11.39)	10.33 (9.94; 10.93)	1.00

### 3.2 Histology

**FIGURE 4:**
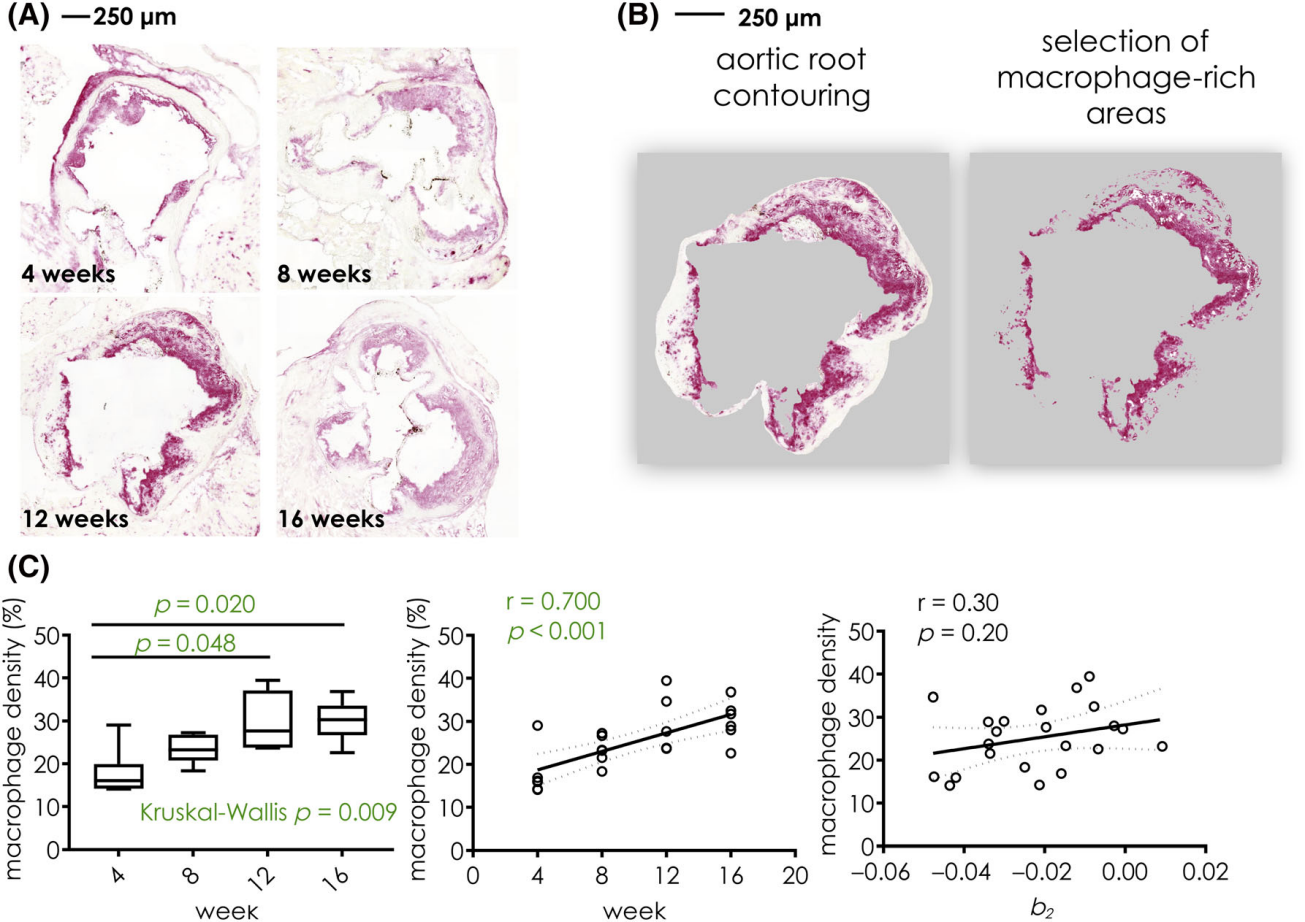
(A) Representative CD68 staining of aortic root of mice at 4 (top left), 8 (top right), 12 (bottom left), and 16 (bottom right) weeks on a high‐fat diet. (B) Quantification of the macrophage percentage area: the inner and outer contours of the aortic root are delineated (left panel); macrophage‐rich areas are then selected using a semiautomated threshold (right panel); macrophage density is calculated as the ratio between macrophage‐rich areas and the whole vessel wall area and is expressed as a percentage (%). (C) Boxplot (left) and linear regression plot (middle) of macrophage density in mice 4, 8, 12, and 16 weeks on a high‐fat diet; the right panel shows a linear regression plot of macrophage density for each animal against the parameter *b*_*2*_ from dynamic contrast‐enhanced magnetic resonance imaging. Midlines in boxplots represent the median, while whiskers represent the minimum and maximum datapoints

Figure 4A shows representative images of CD68 macrophage staining for mice from different groups. Overall plaque area and macrophage‐rich areas increase as atherosclerosis progresses. In Figure 4B we show an example of the aortic root vessel wall contouring (left), together with macrophage‐rich areas (right) identified by semiautomated thresholding. Macrophage density was calculated as the ratio between macrophage‐rich areas and the vessel wall area and is expressed as a percentage.

As shown in Figure 4C, macrophage density significantly increases in mice fed a high‐fat diet for longer periods of time (Kruskall‐Wallis, *p* = 0.009). Between‐groups comparison revealed significant differences between mice fed a high‐fat diet for 4 versus 12 weeks (*p* = 0.048), and 4 versus 16 weeks (*p* = 0.020). Spearman correlation revealed a significant association between the number of weeks on a high‐fat diet and macrophage density (*p* < 0.001, r = 0.70).

Additionally, the relationship between the parameter *b*_*2*_ from DCE‐MRI and macrophage density for each mouse was explored using Spearman correlation. This analysis showed only a limited relationship between these two variables (r = 0.28, *p* = 0.20).

## 4 DISCUSSION

In this study we describe the application of a novel self‐gated DCE‐MRI acquisition with CS reconstruction to study the longitudinal changes in endothelial permeability in the aortic root of atherosclerotic Apoe^−/−^ mice.

Our findings indicate that the contrast agent washout rate from aortic plaques of mice becomes significantly slower as mice are kept on a high‐fat diet for longer periods of time (Kruskal‐Wallis, *p* = 0.03). These in vivo results are mirrored by ex vivo histological findings, which indicate a significant progressive increase in plaque macrophage density from mice kept on a high‐fat diet only for 4 weeks before euthanasia, to animals kept on a high‐fat diet for 8, 12, and 16 weeks (Kruskal‐Wallis, *p* = 0.009). Spearman correlation between macrophage density and the contrast agent washout rate in each animal did not achieve significance (*p* = 0.20, r = 0.3).

Other authors have applied quantitative contrast‐enhanced MR imaging methods to measure endothelial permeability in the atherosclerotic mouse vasculature. For example, Phinikaridou et al. reported the use of an ECG‐triggered Look‐Locker sequence to map R1 changes in the brachiocephalic artery of atherosclerotic mice after the injection of an intravascular contrast agent (gadofosveset trisodium).^24^, ^25^ Using this method the authors demonstrated an increased R1 relaxation rate during atherosclerosis progression^25^ in the brachiocephalic artery and in NOS3−/− mice after arterial denudation,^26^ while lower contrast agent accumulation was observed in wild‐type and in mice treated with statins, minocycline (an antibiotic), and ebselen (a glutathione peroxidase mimetic that has been shown to decrease atherosclerotic burden in several animal models).^24^, ^25^, ^26^ In vivo differences in R1 were mirrored by ex vivo changes in endothelial permeability evaluated using Evans Blue dye. While allowing for absolute R1 quantification, the ECG‐triggered Look‐Locker approach used in these studies is still quite time consuming. To overcome this limitation, Bar et al.^27^ proposed the use of a 3D self‐gated FLASH sequence to quantify R1 changes in the mouse brachiocephalic artery using the variable flip angle method. Using this method, the authors demonstrated increased endothelial permeability in the brachiocephalic artery of atherosclerotic mice, accompanied by an impaired vasodilatory response following administration of acetylcholine. This approach has also been used to quantify cardiac perfusion in mice after myocardial infarction.^28^, ^29^

The current study differs from these approaches in several aspects. Plaque R1 values quantified only at a specific time point after contrast agent injection are not exclusively dependent on endothelial permeability, but also on the distribution volume of the contrast agent in the vessel wall (such as the extracellular matrix). The true quantification of endothelial permeability and other relevant parameters such as microvascular volume, or the extravascular extracellular matrix, requires sampling the full kinetics of tissue contrast agent uptake and washout, which can only be achieved using DCE‐MRI, as proposed in this study. This becomes particularly relevant when investigating the use of small extravascular extracellular agents, such as Gd‐DTPA used in this study, as opposed to the larger, albumin‐binding agents used in the studies mentioned above. While capturing the fast uptake dynamics of small Gd‐based agents may be more challenging, the translational impact of studies performed with these contrast agents may be more prominent, as they are commonly used also in humans. On the contrary, many intravascular agents (such as gadofosveset trisodium) are not available for clinical use anymore or were developed exclusively for preclinical studies.

In addition to our study, other groups have explored options for accelerated cardiovascular DCE‐MRI in small animals,^30^ although mainly with applications in the heart. Very rapid, ECG‐triggered acquisitions have been successfully tested in the past for myocardial perfusion in the mouse.^31^, ^32^, ^33^ However, these approaches did not contemplate black blood imaging, which is preferable for vascular DCE‐MRI, and employed lower spatial resolution with respect to what is required for aortic root imaging. Iterative reconstructions have been previously used to further accelerate DCE‐MRI acquisitions in the mouse. For example, a radial k‐space sampling together with CS Golden‐angle RAdial Sparse Parallel (GRASP) reconstruction has been used for accelerated DCE‐MRI of the mouse brain.^34^ A similar approach, also based on radial k‐space sampling together with a low‐rank “multitasking” reconstruction, has also been proposed for dynamic quantitative T1 mapping of the human carotid arteries.^35^ However, these approaches did not include black blood imaging and employed lower spatial resolution with respect to what is required for aortic root imaging. On the contrary, in our study, we propose to use a black blood based on a modified k‐space matrix with Cartesian sampling for DCE‐MRI of the mouse aortic root. Coupled with an iterative CS reconstruction, our approach provides adequate spatio‐temporal resolution and vessel wall delineation to obtain four‐dimensional data to characterize contrast agent uptake in this vascular structure over time, and in different phases of the cardiac cycle.

However, our approach also has limitations. Firstly, while black blood dynamic imaging allows for better vessel wall delineation compared with bright blood acquisitions, it does not allow performing kinetic modeling for the true quantification of endothelial permeability because contrast agent kinetics in the blood plasma (the so‐called AIF) cannot be reliably sampled.^36^ Therefore, in our case a piecewise curve was used to extract parameters from DCE‐MRI data.^11^ Other possible approaches to overcome this obstacle are the calculation of nonmodel‐based parameters such as the area under the contrast agent concentration curve (AUC), uptake slope, time‐to‐peak or the use of relative kinetic models, which calculate kinetic parameters in the target tissue relative to contrast agent uptake in a reference tissue (such as skeletal muscle),^37^ or the use of dynamic curves classification strategies.^38^ Lastly, our image acquisition encompassed only one slice. This strategy was chosen to allow for sufficiently high temporal resolution to capture contrast agent kinetics, together with adequate spatial resolution for imaging of the small mouse aortic root, but may, however, render the comparison with histology or other ex vivo assays quite challenging.

## 5 CONCLUSIONS

In conclusion, we present the application of a self‐gated DCE‐MRI acquisition with CS reconstruction to quantify longitudinal changes in endothelial permeability during natural progression in a mouse atherosclerosis model. Our results show that the contrast agent washout rate from atherosclerotic plaque is significantly different in mice kept on a high‐fat diet for different lengths of time. In the future, we foresee that further development of this technique from single‐slice to 3D slab coverage in the aortic root and ascending aorta will allow better investigation of the relationship between in vivo imaging of endothelial permeability and atherosclerotic plaques’ genetic, molecular, and cellular makeup in this important model of disease.

## CONFLICTS OF INTEREST

The authors declare no competing interests.
